# The Science of Style: In Fashion, Colors Should Match Only Moderately

**DOI:** 10.1371/journal.pone.0102772

**Published:** 2014-07-17

**Authors:** Kurt Gray, Peter Schmitt, Nina Strohminger, Karim S. Kassam

**Affiliations:** 1 Department of Psychology, University of North Carolina, Chapel Hill, North Carolina, United States of America; 2 Department of Psychology, Duke University, Durham, North Carolina, United States of America; 3 Department of Social and Decision Sciences, Carnegie Mellon University, Pittsburgh, Pennsylvania, United States of America; University of New South Wales, Australia

## Abstract

Fashion is an essential part of human experience and an industry worth over $1.7 trillion. Important choices such as hiring or dating someone are often based on the clothing people wear, and yet we understand almost nothing about the objective features that make an outfit fashionable. In this study, we provide an empirical approach to this key aesthetic domain, examining the link between color coordination and fashionableness. Studies reveal a robust quadratic effect, such that that maximum fashionableness is attained when outfits are neither too coordinated nor too different. In other words, fashionable outfits are those that are moderately matched, not those that are ultra-matched (“matchy-matchy”) or zero-matched (“clashing”). This balance of extremes supports a broader hypothesis regarding aesthetic preferences–the Goldilocks principle–that seeks to balance simplicity and complexity.

## Introduction

Every day, people ask themselves the question: “What to wear?” People want outfits that are maximally fashionable, and this isn’t mere vanity: clothing influences perceived and signaled social identity [1], employment outcomes [2], romantic success [3], and even cognitive processes [4]. Despite its universal human importance and vast financial worth–the fashion industry is valued at $1.7 trillion (more than double the entire U.S. federal science budget) –there is little empirical psychological research on the objective features which make something fashionable. In this study, we provide an empirical approach to fashionableness, through judgments of color combinations. We uncover practical implications for daily life, and in doing so speak to a broader theory in aesthetics and human preferences–the Goldilocks Principle.

The Goldilocks Principle represents a tradition of philosophical thought stretching back millennia: Aristotle’s Golden mean, Buddha’s middle way, and Confucius’ Doctrine of the Mean all represent a balance between two extremes. The Goldilocks Principle has psychological support in a variety of domains, as infants prefer looking at visual sequences that are neither too complex, nor too simple [5], and optimum psychological well-being–i.e., flow–is achieved when experiences balance simplicity and complexity [6]. The optimal distinctiveness model of social identity suggests that when developing a sense of self, we strive to strike a harmonious balance between similarity with others and individual distinctiveness [7]. Furthermore, judgments of facial attractiveness across cultures are predicted by averageness [8] [9], suggesting that the aesthetic ideal is found not at the extremes, but rather in balance.

In terms of fashion, there are two popular approaches to style that represent “extremes.” On one hand, we often speak as if the most fashionable outfits are those that fully coordinate or “match” [10]. This suggests that pairing the same or similar colors with each other may be the key to fashion. On the other hand, fashion is often about being noticed, and so we might want color combinations that maximally differ from each other and “pop” [11]. Between these two extremes, the Goldilocks Principle suggests that the best color combinations are those that are neither too similar (“matchy-matchy”) nor too different (“clashing”).

In this paper, we investigate whether the Goldilocks Principle predicts fashionableness across diverse color combinations in both men’s and women’s outfits. Support for the principle would be illustrated by a “peak” in ratings, such that any linear trends between coordination and fashionableness should be qualified by a quadratic effect such that maximum fashionableness is achieved by moderate color coordination.

## Method

**Figure 1:**
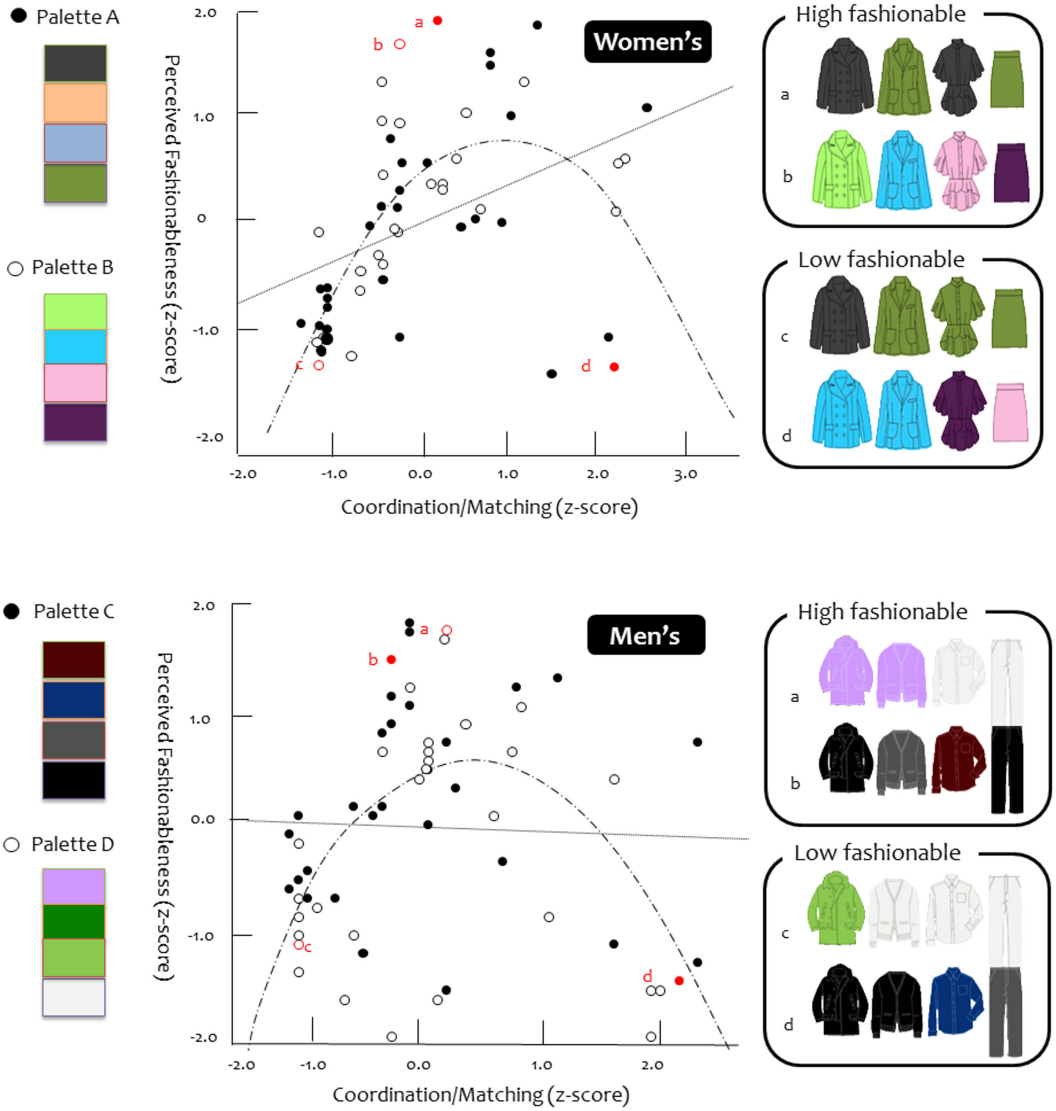
Coordination/Matching (z-score).

This study was approved by the Institutional Review Board at The University of North Carolina at Chapel Hill. All participants provided written informed consent prior to completing the study and were recruited through Amazon’s Mechanical Turk (mTurk). A total of 239 mTurk participants (69% women, *M_age_* = 35.4, *SD*_age_ = 12.9) each saw 30 different color combinations, in one of four color palettes. Palettes 1 and 2 were in women’s clothing, and palettes 3 and 4 were in men’s clothing. Each palette included 4 colors, illustrated in Figure 1. Out of 256 possible color combinations within each set, we selected combinations quasi-randomly to represent a range of coordination, from all matching to all different (see Figure 1 for sample outfits, Table S1 for all outfit combinations, Figure S1 for screen shots of fashionableness, and Figure S2 for screen shots of coordination judgments).

### Fashionableness Judgments

Participants rated clothing sets on how fashionable, good, and liked they were on five point Likert scales. Ratings were aggregated across participants to yield an overall fashionableness rating for each combination (all αs >.95), which were Z-scored within each palette.

### Coordination Calculations

Rather than solicit global ratings of coordination, participants rated the coordination of all possible pairs of color swatches within each palette through 3 items–coordinated, matching and similar–answered on five point Likert scales (α’s >.81). These pairwise judgments were aggregated for each outfit, and Z-scored within each palette, to create an overall coordination score. Thus, combinations with all similar or highly coordinated colors (e.g., only shades of green) received high scores whereas those with very different colors (e.g., red, blue, black, grey) received low scores (see Materials and Methods S1 for additional detail on these calculations).

### Analysis

Curve estimation was used to assess linear and quadratic effects of coordination on fashionableness. Women’s and men’s clothing were analyzed separately.

## Results

### Women’s Clothing

Analyses revealed a significant linear trend, *R* ^2^ = .18, *F* (1, 58) = 13.04, *p* = .001, such that more coordination was linked to more fashionableness, consistent with the general importance of matching. Importantly, however, this linear trend was qualified by the predicted quadratic effect, *R* ^2^ = .44, *F* (2, 57) = 22.23, *p*<.001, such that peak fashionableness was achieved by moderately coordinated combinations. This quadratic effect accounted for twice as much variance as the linear effect.

### Men’s Clothing

Analyses did not reveal a significant linear trend, *F*<1, but did reveal the predicted quadratic trend, *R* ^2^ = .28, *F* (2, 57) = 11.18, *p*<.001, such that peak fashionableness was again achieved by moderately coordinated combinations.

## Discussion

These data suggest a simple answer to the question “what to wear?” Select a color combination that is neither completely uniform, nor completely different. Certainly, moderate matching is not the only key to fashion, which varies across time and culture and depends upon many factors including cut, design, and trendiness. However, these studies reveal that, with all other factors held constant, the Goldilocks principle predicted judgments across four different color palettes in both men’s and women’s clothing. To examine the external validity of these findings, future research should test this idea in naturalistic settings, such as in magazines and runway shows.

These results are consistent with both centuries of philosophical thought and more recent psychological studies on the importance of “the middle way.” The Goldilocks principle may also explain aesthetic judgments beyond fashion, reflecting a basic principle of human preference that seeks to balance simplicity and complexity, order and disorder. Indeed, people prefer music that balances melodic simplicity and complexity [12]. This quantitative analysis of fashion is only a first step in empirical aesthetics, but it highlights the utility of bringing science to art; psychological science can help explain the important but often invisible judgments of daily life.

## Supporting Information

Figure S1 **Rating a sample outfit combination.**
(DOCX)

Figure S2 **Rating a sample color pair.**
(DOCX)

Table S1 **Clothing combinations by color palette.**
(DOCX)

Table S2 **Cronbach’s Alphas for scales used.**
(DOCX)

Materials and Methods S1 **Extended materials & methods.**
(DOCX)

Results S1 **Extended results.**
(DOCX)
