# Supplementary figures and images for: The Science of Style: In Fashion, Colors Should Match Only Moderately
Source: PLoS One. 2014 Jul 17;9(7):e102772. doi: 10.1371/journal.pone.0102772 (PMC4102554; doi:10.1371/journal.pone.0102772)

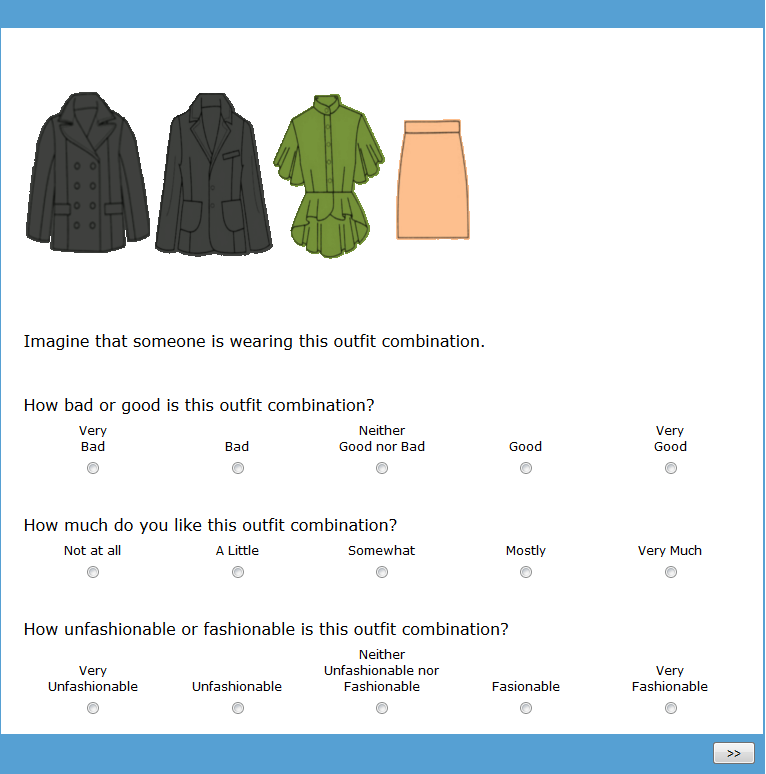


*Figure S1.* Rating a sample outfit combination.

Supplement: Figure S1 — Rating a sample outfit combination. (DOCX) [file pone.0102772.s001.docx]

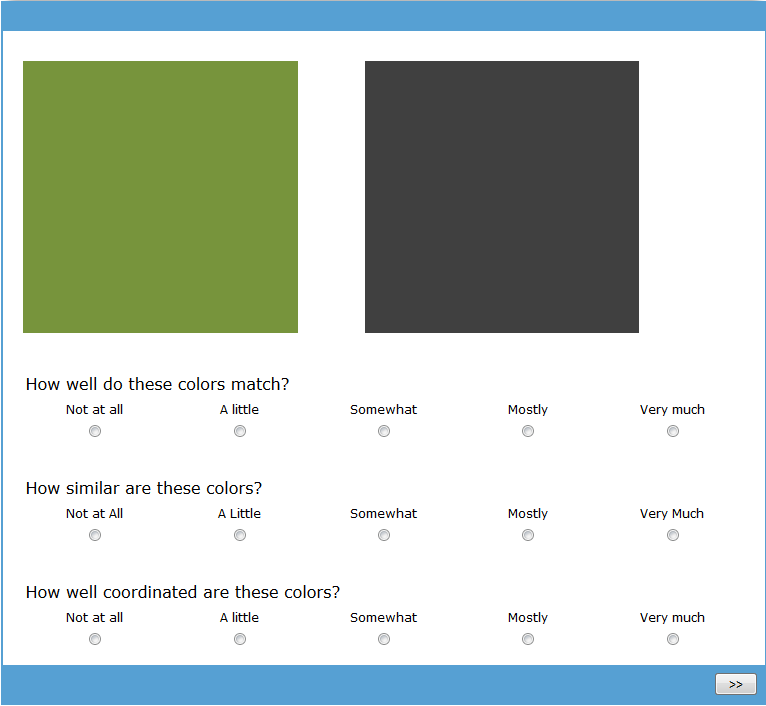


*Figure S2*. Rating a sample color-pair.

Supplement: Figure S2 — Rating a sample color pair. (DOCX) [file pone.0102772.s002.docx]

Table S1. Clothing combinations by color palette

| Outfit | Palette 1 | Palette 2 | Palette 3 | Palette 4 |
| --- | --- | --- | --- | --- |
| 1 | 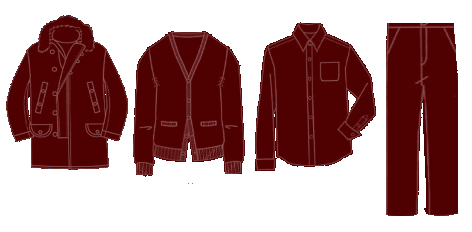 | 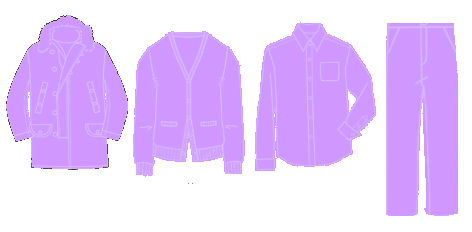 | 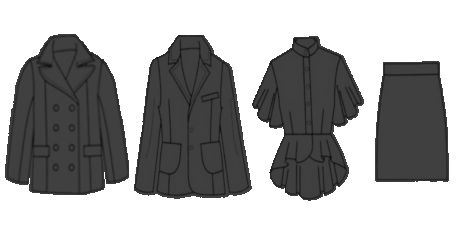 | 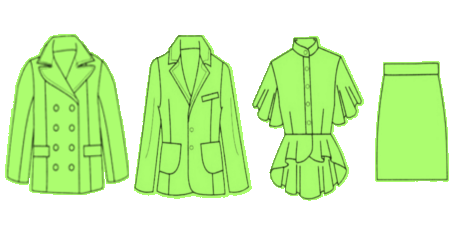 |
| 2 | 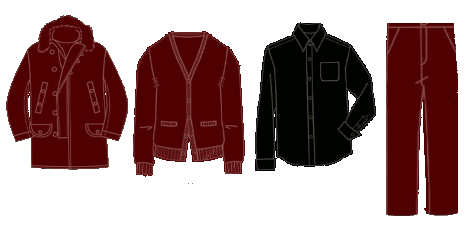 | 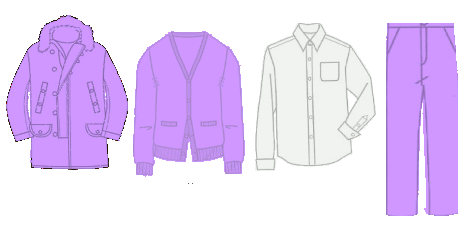 | 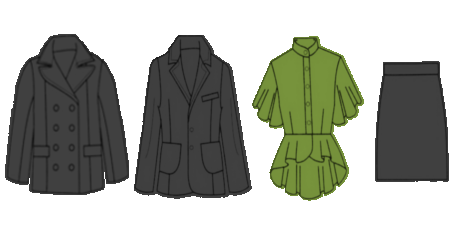 | 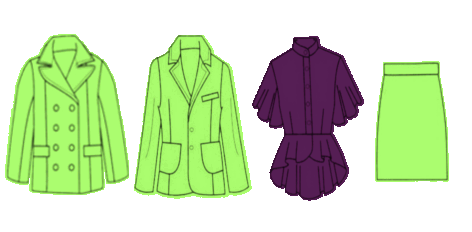 |
| 3 | 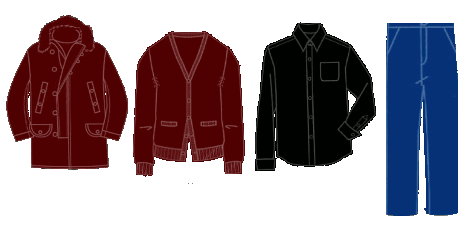 | 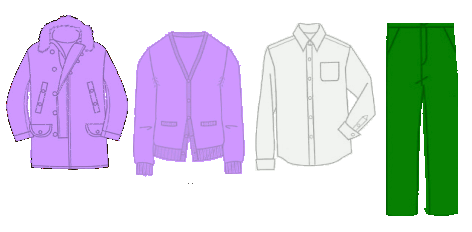 | 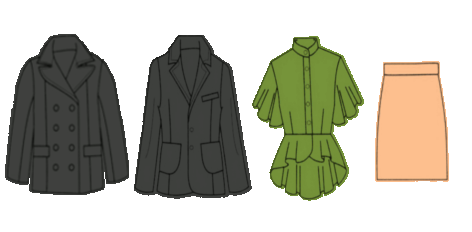 | 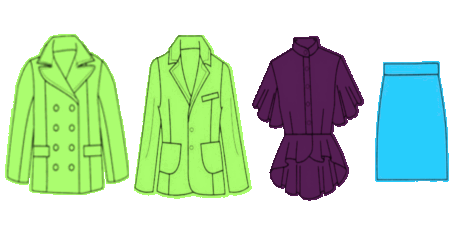 |
| 4 | 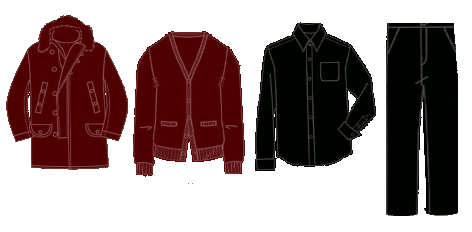 | 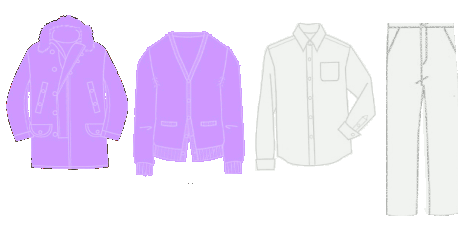 | 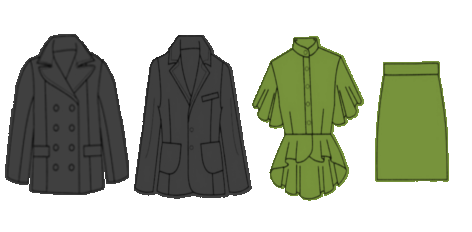 | 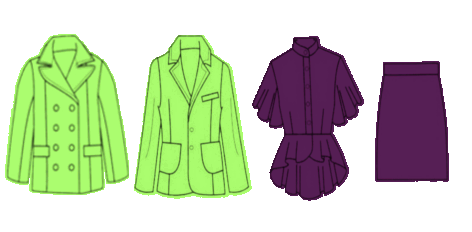 |
| 5 | 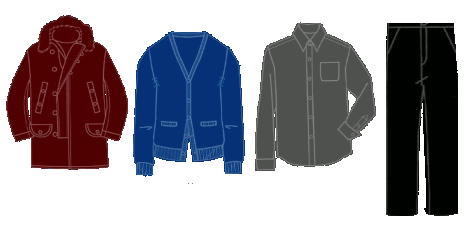 | 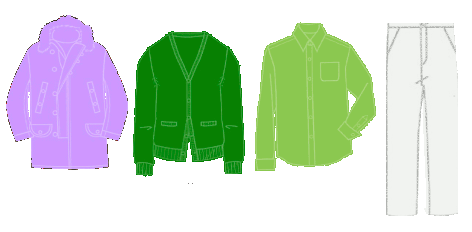 | 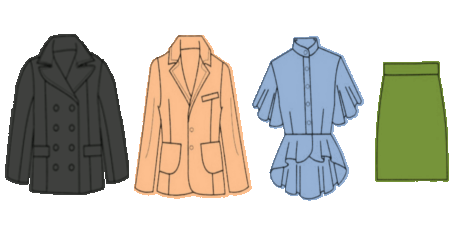 | 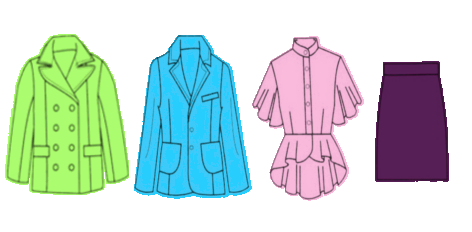 |
| 6 | 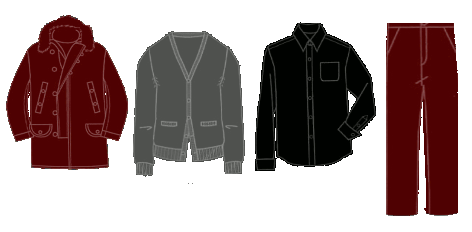 | 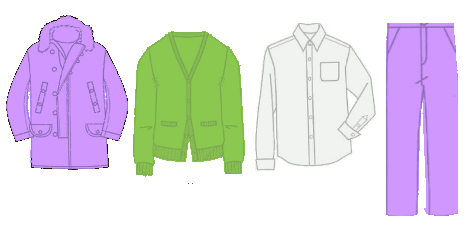 | 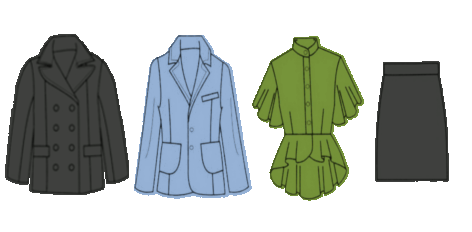 | 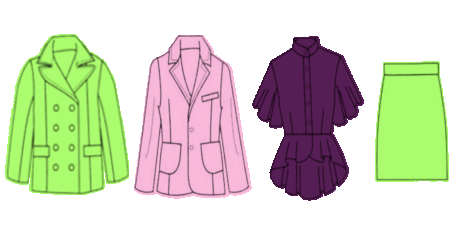 |
| 7 | 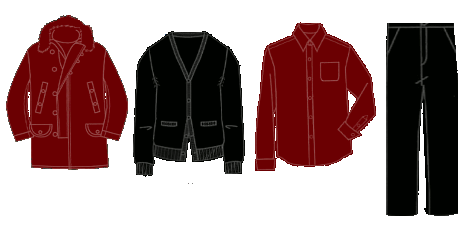 | 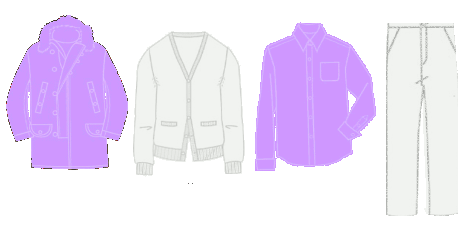 | 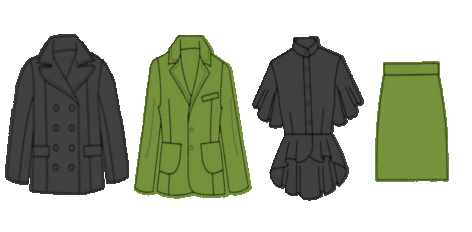 | 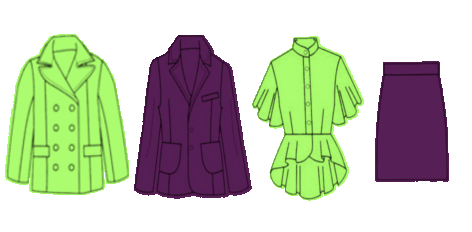 |
| 8 | 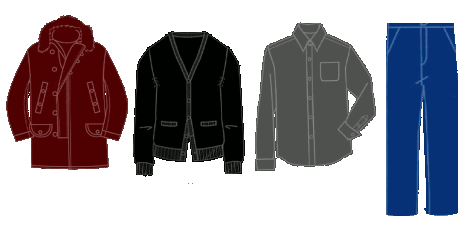 | 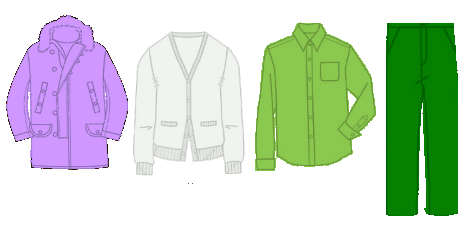 | 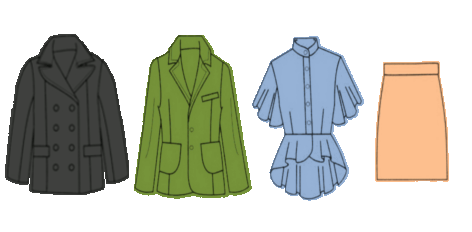 | 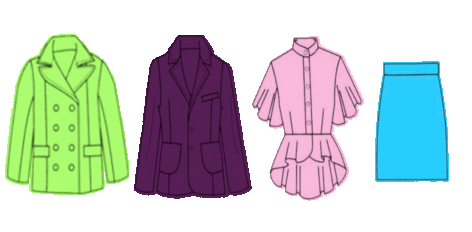 |
| 9 | 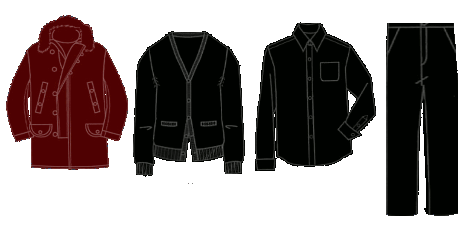 | 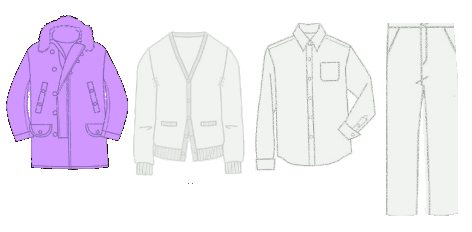 | 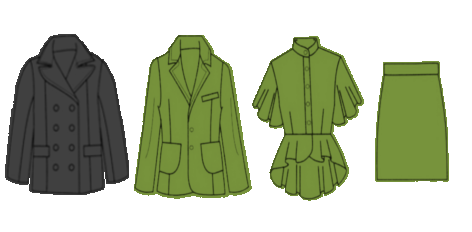 | 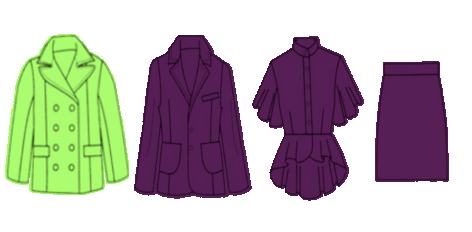 |
| 10 | 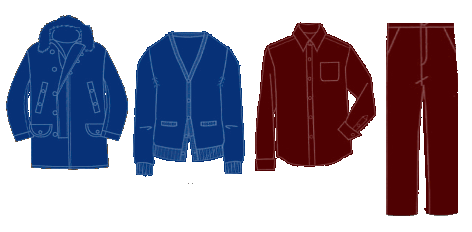 | 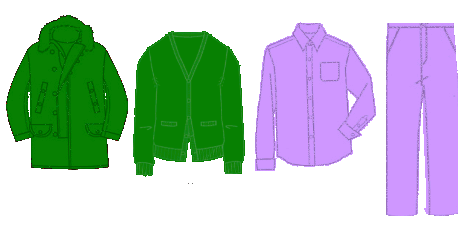 | 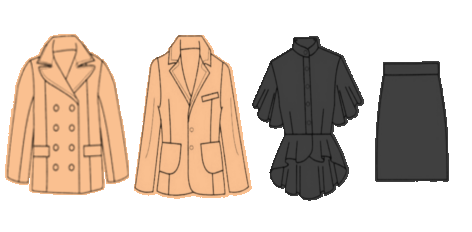 | 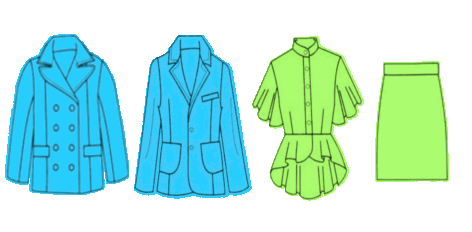 |
| 11 | 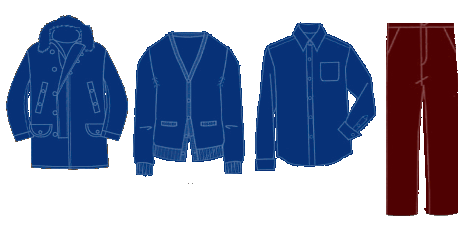 | 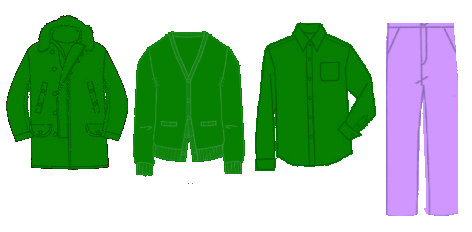 | 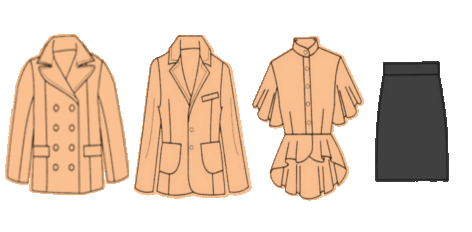 | 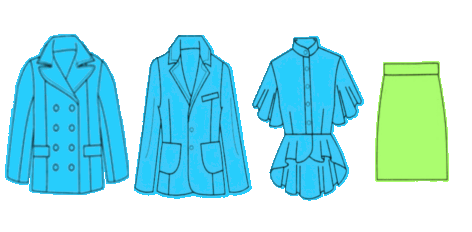 |
| 12 | 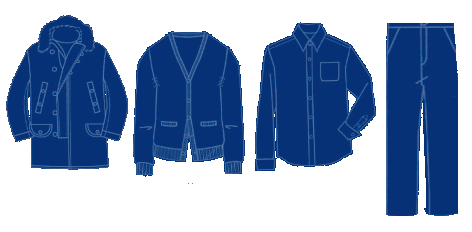 | 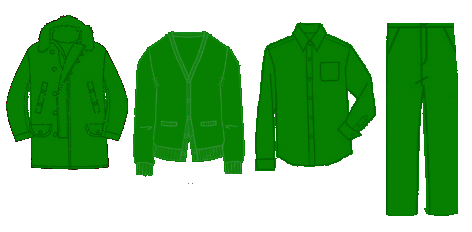 | 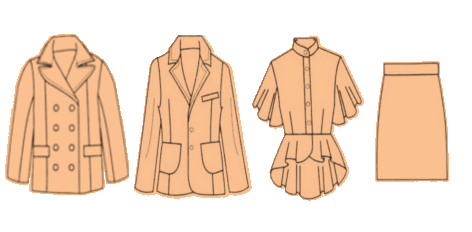 | 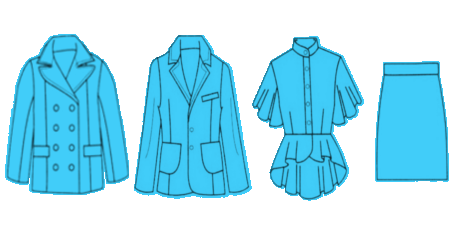 |
| 13 | 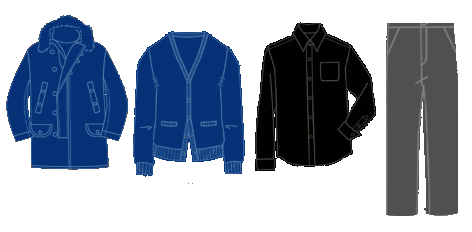 | 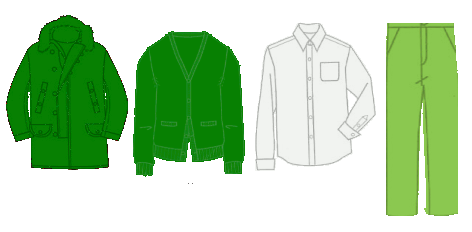 | 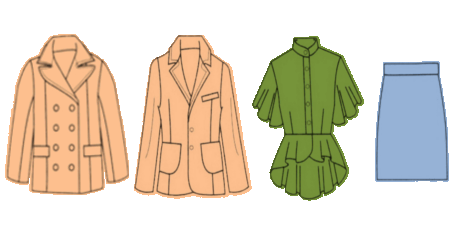 | 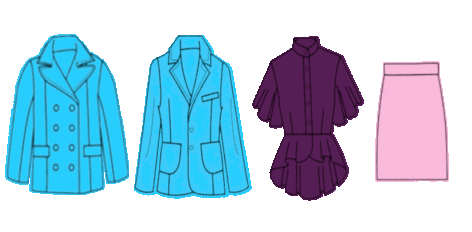 |
| 14 | 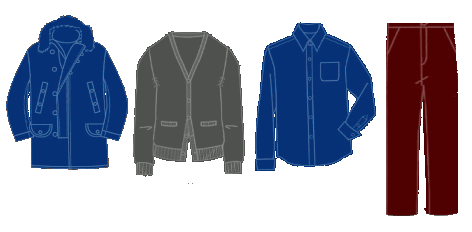 | 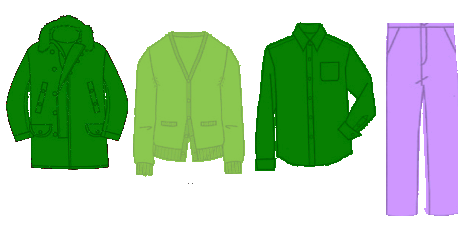 | 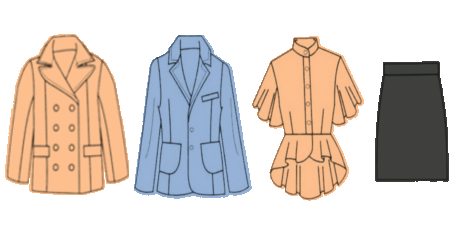 | 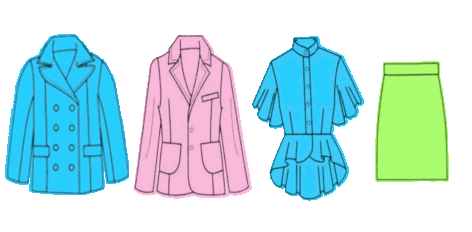 |
| 15 | 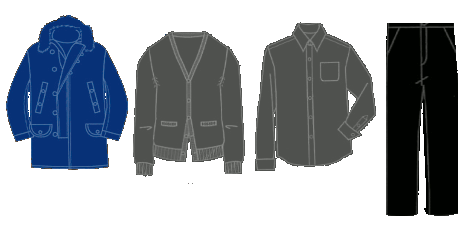 | 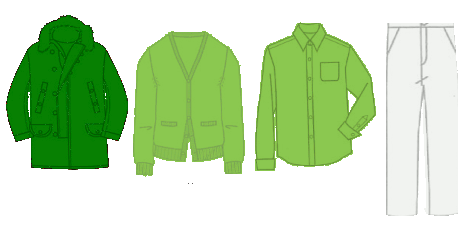 | 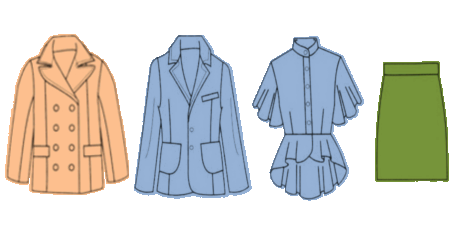 | 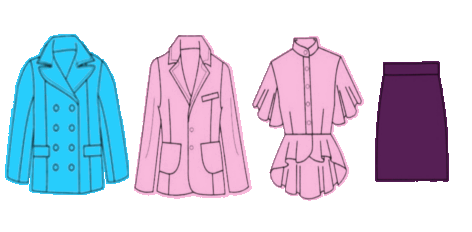 |
| 16 | 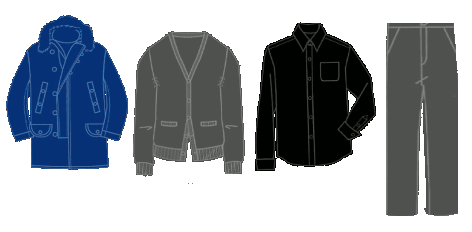 | 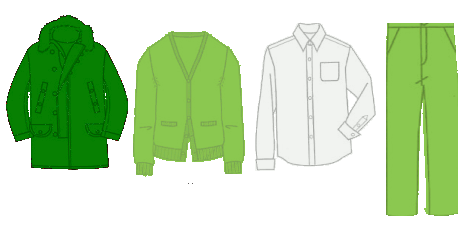 | 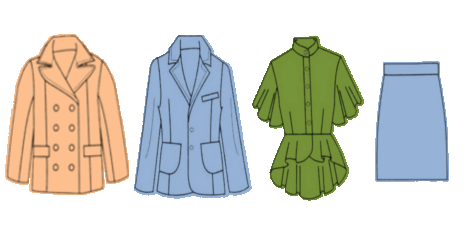 | 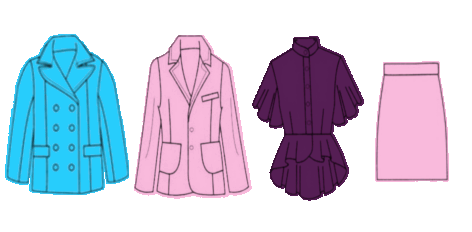 |
| 17 | 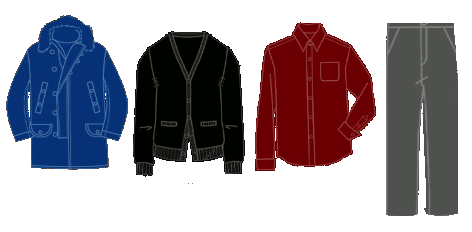 | 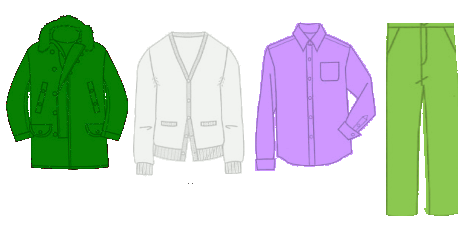 | 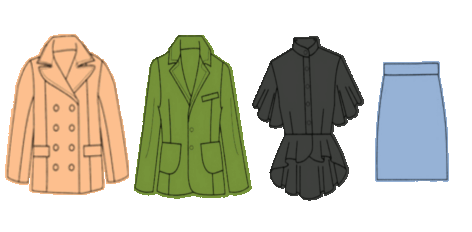 | 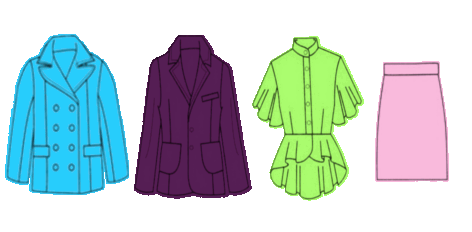 |
| 18 | 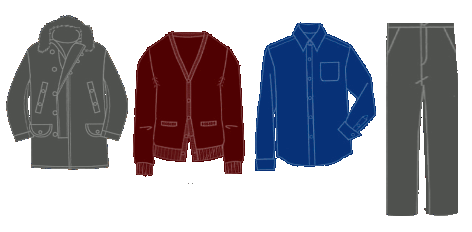 | 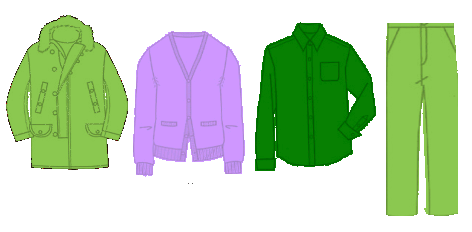 | 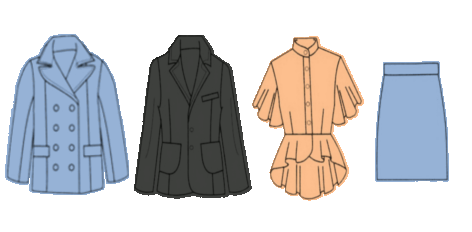 | 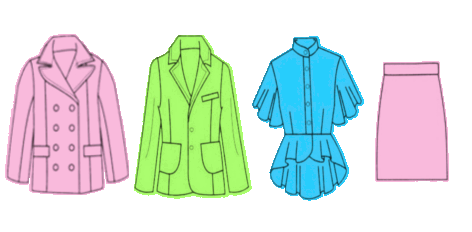 |
| 19 | 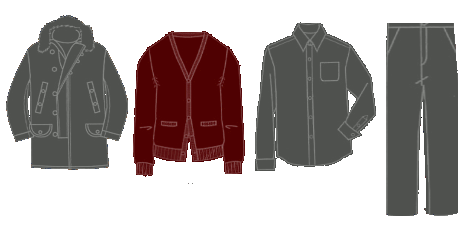 | 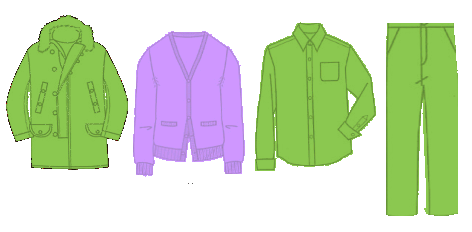 | 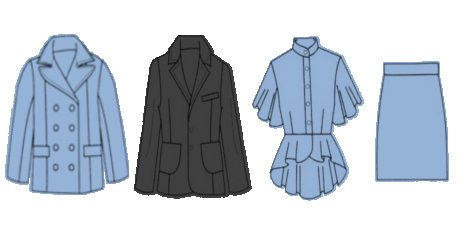 | 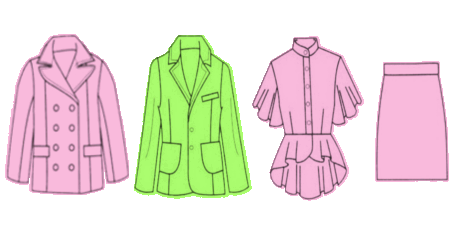 |
| 20 | 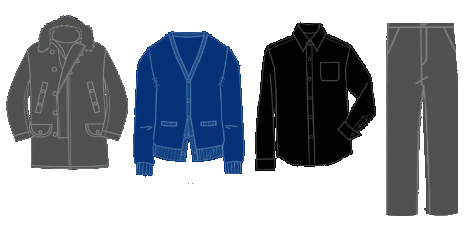 | 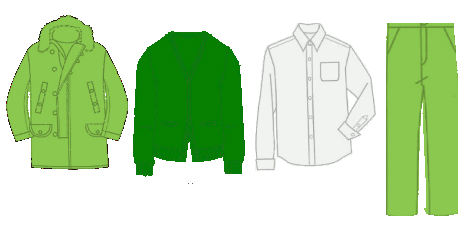 | 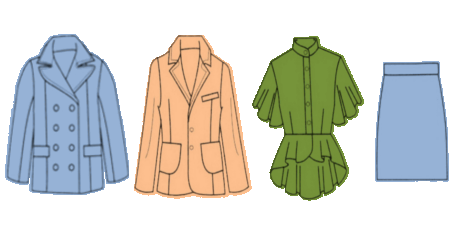 | 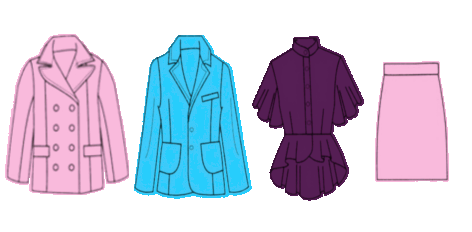 |
| 21 | 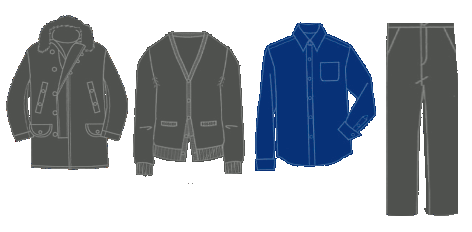 | 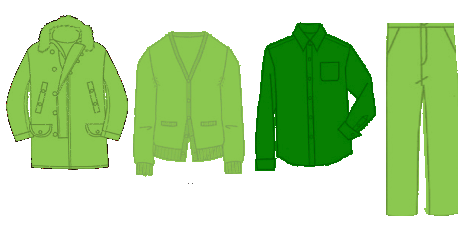 | 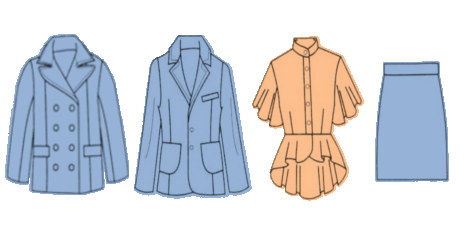 | 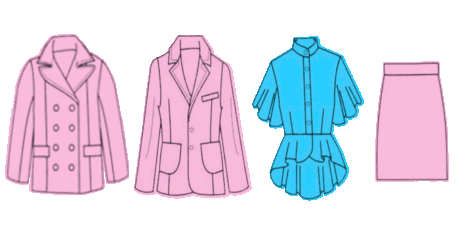 |
| 22 | 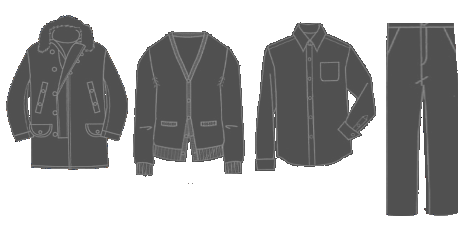 | 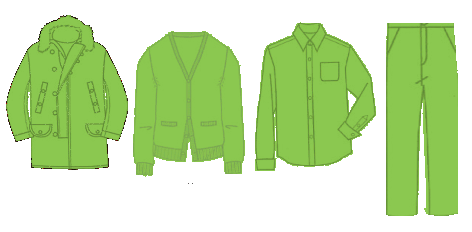 | 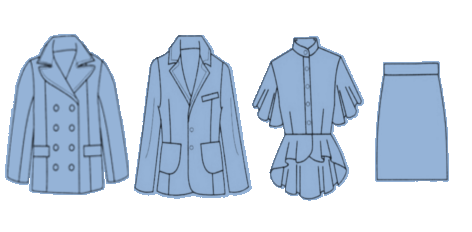 | 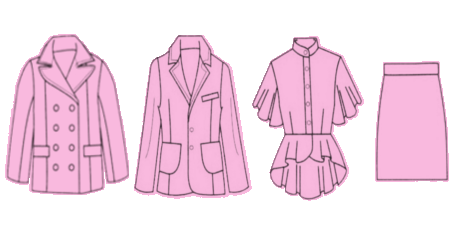 |
| 23 | 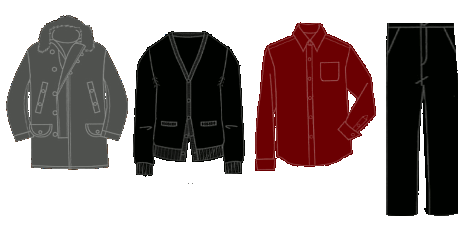 | 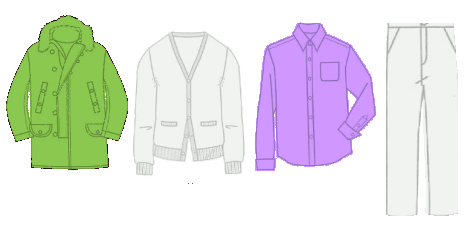 | 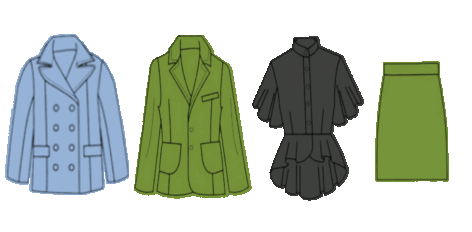 | 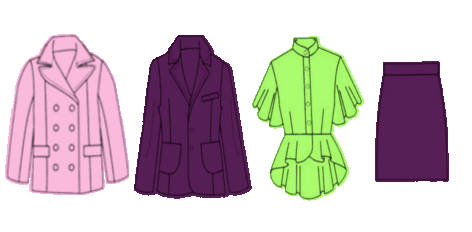 |
| 24 | 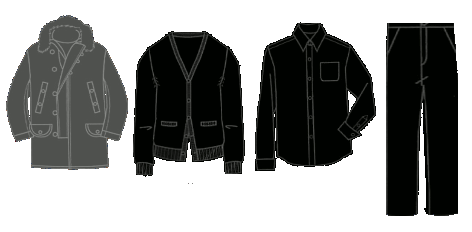 | 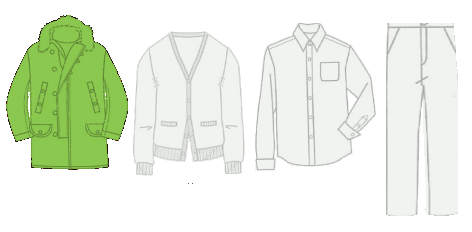 | 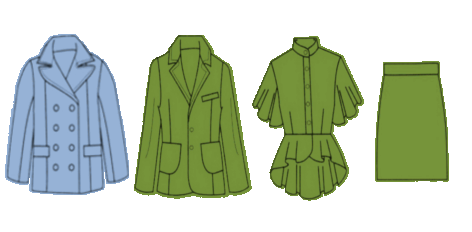 | 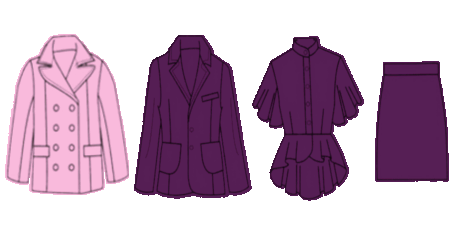 |
| 25 | 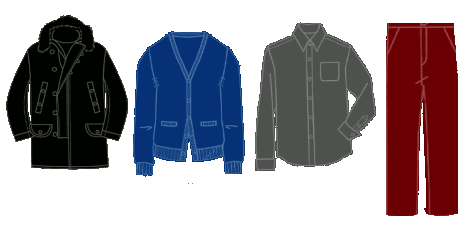 | 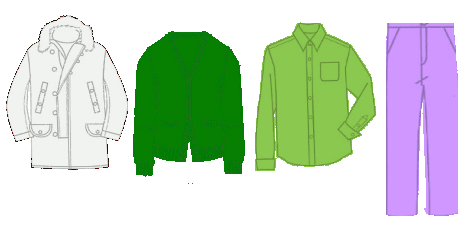 | 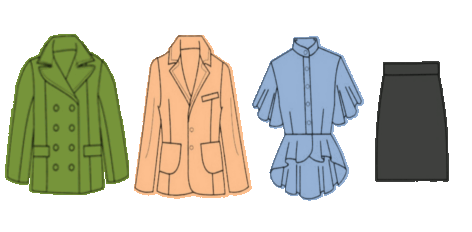 | 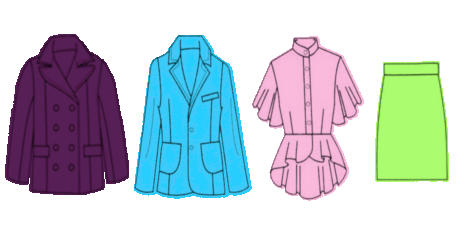 |
| 26 |  |  |  |  |
| 27 |  |  |  |  |
| 28 |  |  |  |  |
| 29 |  |  |  |  |
| 30 |  |  |  |  |

Supplement: Table S1 — Clothing combinations by color palette. (DOCX) [file pone.0102772.s003.docx]
