# Supplementary material for: The Science of Style: In Fashion, Colors Should Match Only Moderately
Source: PLoS One. 2014 Jul 17;9(7):e102772. doi: 10.1371/journal.pone.0102772 (PMC4102554; doi:10.1371/journal.pone.0102772)
Supplement: Table S2 — Cronbach’s Alphas for scales used. (DOCX) [file pone.0102772.s004.docx]

Table S2. Alphas

|  | Cronbach’s Alphas |  |
| --- | --- | --- |
|  | Fashion | Coordination |
| Palette 1 | .95 | .81 |
| Palette 2 | .95 | .83 |
| Palette 3 | .94 | .81 |
| Palette 4 | .95 | .86 |
