# Supplementary material for: The Science of Style: In Fashion, Colors Should Match Only Moderately
Source: PLoS One. 2014 Jul 17;9(7):e102772. doi: 10.1371/journal.pone.0102772 (PMC4102554; doi:10.1371/journal.pone.0102772)
Supplement: Materials and Methods S1 — Extended materials & methods. (DOCX) [file pone.0102772.s005.docx]

**Materials and Methods**

**Participants**

**Palette 1.** A total of 100 participants (69% women, *M_age_* = 37.25, *SD* = 12.94, age range 20-73 years) saw 30 outfits of different color combinations, consisting of blue, black, grey, and red. Participants self-reported how fashionable they were, how much thought they put into their dress, how up to date they are on trends, and how out of fashion they are, all on 7-point Likert scales. These ratings were combined into an index of fashionableness (α = .71, *M* = 4.23, *SD* = 1.07). 99% of participants reported their nationality as American. 1 participant reported their nationality as Indian. Participants provided ratings of their political orientation on a 7 point scale, from “strongly liberal” (1) to “strongly conservative” (7). Average political orientation was moderate (*Mdn* = 4.00, *M* = 3.51, *SD* = 1.82).

**Palette 2.** A total of 43 participants (67% women, *M_age_* = 33.65, *SD* = 10.04, age range 20-63 years) saw 30 outfits of different color combinations, consisting of pink, green, light green, and white. Participants reported their own fashionableness on the same 4 items, which were then combined into an index of fashionableness (α = .71, *M* = 4.45, *SD* = 1.07). 51% of participants reported their nationality as American. 1 participant reported their nationality as Argentinian, 1 as Bulgarian, 1 as Chinese, 1 as Finnish, 1 as Ghanaian, 1 as New Zealander, 3 as Filipino, 1 as Romanian, 1 as Russian, 3 as Serbian, 2 as Macedonian, and 1 as British. Average political orientation was somewhat liberal (*Mdn* = 3.00, *M* = 3.28, *SD* = 1.37).

**Palette 3.** A total of 41 participants (61% female, *M_age_* = 32.07, *SD* = 13.55, age range 19-80 years) saw 30 outfits of different color combinations, consisting of black, tan, blue, and green. Participants reported their own fashionableness on the same 4 items, which were then combined into an index of fashionableness (α = .81, *M* = 4.76, *SD* = 1.08). 85% of participants reported their nationality as American. 1 participant reported their nationality as Bulgarian, 1 as Canadian, 1 as German, 2 as Serbian, and 1 as British. Average political orientation was moderate (*Mdn* = 4.00, *M*  = 3.61, *SD* = 1.80).

**Palette 4.** A total of 45 participants (58% female, *M_age_* = 34.56, *SD* = 12.28, age range 20-66 years) saw 30 outfits of different color combinations, consisting of green, blue, pink and purple. Participants reported their own fashionableness on the same 4 items, which were then combined into an index of fashionableness (α = .62, *M* = 4.63, *SD* = .92). 78% of participants reported their nationality as American. 1 participant reported their nationality as Bulgarian, 2 as Canadian, 1 as Chilean, 1 as Indonesian, 1 as Jamaican, 1 as Pakistani, 1 as Serbian, and 1 as Turkish. Average political orientation was moderate (*Mdn* = 4.00, *M* = 3.71, *SD*  = 1.82).

**Stimuli**

30 outfit combinations were presented to participants (see Table S1). In palettes 1 and 2 (men’s), outfits consisted of a jacket, cardigan, shirt and pants. In palettes 3 and 4 (women’s), outfits consisted of a pea coat, blazer, blouse, and skirt.

**Experimental Paradigm**

After providing consent, participants were presented with individual outfit combinations. Outfits were presented in a randomized order. Color combinations were quasi-randomly assigned, and remained consistent across palettes. Ratings were made of how good, liked, and fashionable each outfit was on five-point scales (see Figure S1). These ratings were aggregated across participants, providing an overall fashionableness rating for each combination (see Table S2 for a breakdown of α’s by color palette). These ratings were then Z-scored within each palette.

Participants then provided ratings of individual color combinations, resulting in 10 ratings within each palette (see Figure S2). Ratings were made of how matched, similar, and coordinated each outfit was on five-point scales. These ratings were indexed into a measure of coordination that was found to be highly reliable (see Table S2 for a breakdown of α’s by color palette). This measure was then Z-scored within each palette, providing Z-scores for each individual color combination (e.g., blue-red, blue-blue, black-grey). As each outfit consisted of 4 clothing items, each contained 6 individual color combinations (e.g., jacket-shirt, jacket-pants, shirt-pants, etc.). The Z-scores of each color combination were summed for each outfit, providing an overall coordination score for every individual outfit. The resulting sums were transformed into Z-scores of overall coordination.
