# Supplementary material for: The Science of Style: In Fashion, Colors Should Match Only Moderately
Source: PLoS One. 2014 Jul 17;9(7):e102772. doi: 10.1371/journal.pone.0102772 (PMC4102554; doi:10.1371/journal.pone.0102772)
Supplement: Results S1 — Extended results. (DOCX) [file pone.0102772.s006.docx]

**Results**

**Men’s**

**Palette 1.** Analyses did not reveal a significant linear trend, *F* < 1, but did reveal the predicted quadratic trend (*R*^2^ = .25, *F* (2, 27) = 4.51, *p* < .05), such that peak fashionableness was achieved by moderately coordinated combinations.

**Palette 2.** Analyses did not reveal a significant linear trend, *F* < 1, but did reveal the predicted quadratic trend (*R*^2^ = .42, *F* (2, 27) = 9.64, *p* = .001), such that peak fashionableness was achieved by moderately coordinated combinations.

**Women’s**

**Palette 3.** Analyses revealed a marginally significant linear trend (*R*^2^ = .127, *F* (1, 28) = 4.07, *p* = .053), such that more coordination was linked to more fashionableness. This linear trend was qualified by the predicted quadratic trend, (*R*^2^ = .362, *F* (2, 27) = 7.67, *p* < .01).

**Palette 4.** Analyses revealed a significant linear trend (*R*^2^ = .28, *F* (1, 28) = 11.05, *p* < .01), such that more coordination was linked to more fashionableness. This linear trend was qualified by the predicted quadratic trend, (*R*^2^ = .61, *F* (2, 27) = 21.34, *p* < .001).

**Combined**

Analyses revealed a significant linear trend (*R*^2^ = .06, *F* (1, 116) = 7.26, *p* < .01), such that more coordination was linked to more fashionableness. This linear trend was qualified by the predicted quadratic trend, (*R*^2^ = .30, *F* (2, 115) = 24.77, *p* < .001)
